# Genome-wide transcriptomic analysis of the forebrain of postnatal *Slc13a4*^+/−^ mice

**DOI:** 10.1186/s13104-021-05687-5

**Published:** 2021-07-13

**Authors:** Tracey J. Harvey, Raul Ayala Davila, Diana Vidovic, Sazia Sharmin, Michael Piper, David G. Simmons

**Affiliations:** School of Biomedical Sciences, The Faculty of Medicine, The University of Queensland, Brisbane, QLD 4072 Australia

**Keywords:** Forebrain, RNA-sequencing, *Slc13a4*, Sulfate

## Abstract

**Objective:**

Sulfation is an essential physiological process that regulates the function of a wide array of molecules involved in brain development. We have previously shown expression levels for the sulfate transporter *Slc13a4* to be elevated during postnatal development, and that sulfate accumulation in the brains of *Slc13a4*^+/−^ mice is reduced, suggesting a role for this transporter during this critical window of brain development. In order to understand the pathways regulated by cellular sulfation within the brain, we performed a bulk RNA-sequencing analysis of the forebrain of postnatal day 20 (P20) *Slc13a4* heterozygous mice and wild-type litter mate controls.

**Data description:**

We performed an RNA transcriptomic based sequencing screen on the whole forebrain from *Slc13a4*^+/−^ and *Slc13a4*^+/+^mice at P20. Differential expression analysis revealed 90 differentially regulated genes in the forebrain of *Slc13a4*^+/−^ mice (a *p*-value of 0.1 was considered as significant). Of these, 55 were upregulated, and 35 were downregulated in the forebrain of heterozygous mice. Moreover, when we stratified further with a ± 1.2 fold-change, we observed 38 upregulated, and 16 downregulated genes in the forebrain of heterozygous mice. This resource provides a useful tool to interrogate which pathways may require elevated sulfate levels to drive normal postnatal development of the brain.

## Objective

Sulfate is an abundant anion in circulation, and its enzymatic conjugation (sulfation) to a variety of molecules is a biotransformation widely utilised to regulate biological activity [1]. Within the developing brain, sulfation reactions alter the functions of extracellular matrix components, in turn regulating local growth factor interactions critical for neurogenesis or perineuronal net formation [2, 3]. Sulfate is also a substrate for brain cerebrosides and neurotransmitters [1]. To provide sufficient sulfate for these critical sulfation reactions, cells either metabolize sulfur-containing amino acids to release intracellular sulfate, or uptake inorganic sulfate across the plasma membrane via transporters. The maintenance of a low ratio of cerebrospinal fluid (CSF)/serum sulfate level indicates the selective transport of sulfate to maintain brain levels within a tight range [4]. Yet despite its importance, sulfate levels are not typically measured clinically, and therefore the contribution of sulfate deficiencies to neurodevelopmental disorders and disease is poorly appreciated and understood.

Recently, we reported that haploinsufficiency for the sulfate transporter *Slc13a4* resulted in abnormal social behaviours, memory deficits and altered neurogenesis in mice [5, 6]. SLC13A4 is expressed predominantly within the choroid plexus and pia mater of the brain, in an orientation that suggests a role in transporting sulfate from the blood into the CSF [5]. Expression of this transporter therefore appears counter to the notion that sulfate is actively pumped out of the CSF [1]. Nevertheless, *Slc13a4*^+/−^ mice, when injected systemically through the tail vein, accumulate ~ 50% less radiolabelled sulfate within their brains than do *Slc13a4*^+/+^ mice [5]. Fitting with the model of active transport of sulfate out of the CSF, SLC13A4 activity is not essential for adult brain function, as conditional deletion of *Slc13a4* in adult mice does not result in the onset of cellular or behavioural phenotypes. However, deletion of one *Slc13a4* allele in early postnatal development does result in the onset of abnormal social behaviours, memory deficits and increased neurogenesis, suggesting elevated sulfate transport into the brain is critical during early developmental stages of brain formation [5]. Indeed, gene expression analysis indicates that expression of multiple sulfate transporters peak in postnatal mouse development [5], implying a critical role for sulfate availability and metabolism during this developmental window. In line with this, newborn humans have higher serum sulfate levels than children at 3-years of age or adults [7].

The question remains: what critical pathways require elevated sulfate availability during the postnatal period for normal brain development? To address this question, we have undertaken an unbiased RNA-Seq screen to compare the transcriptome of the forebrain of *Slc13a4*^+/−^ mice to control *Slc13a4*^−/−^ littermates at postnatal day 20.

## Data description

The rationale behind this profiling experiment was to understand the differential gene expression that arise during postnatal development in the mouse forebrain when one allele of *Slc13a4* gene is absent. To do this, *Slc13a4*^+/−^ mice and control wild-type littermates were used. These mice were maintained on a C57BL6 background. To generate *Slc13a4*^+/−^ mice, *Slc13a4*^+/−^ male mice were crossed to wild-type female mice. Polymerase chain reaction (PCR) was used to identify the genotype of the offspring (PCR primers are available on request). P20 *Slc13a4*^+/−^ and control mice were cervically dislocated and whole brains were removed and placed on ice. The forebrain was isolated and snap frozen using dry ice. An RNeasy Micro Kit (QIAGEN) was used to extract total RNA from these samples, and 5–10 μg RNA in a total volume of 20 μl was sent to the Institute for Molecular Biosciences Sequencing Facility (The University of Queensland). The sequencing facility assessed sample quality using a Bioanalyzer. All samples passed the quality control with an RNA integrity number > 8. A second analysis was performed to measure the purity of the RNA using a spectrophotometer to determine the OD 260/280 ratio; all samples had values ~ 2.

RNA-Seq libraries were prepared using the Illumina TruSeq Stranded Total RNA LT (Ribo-Zero Gold) Sample Prep Kit (Illumina, RS-122-2301/RS-122-2302), according to the standard manufacturer’s protocol (Illumina, 15031048 Rev. E October 2013) described briefly as follows. To enrich for mRNA, 1 µg of total RNA was depleted of rRNA using Ribo-Zero Gold. The enriched mRNA was then subjected to a heat fragmentation step aimed at producing fragments between 130 and 290 base pairs (average 185 base pairs). cDNA was synthesised from the fragmented RNA using SuperScript II Reverse Transcriptase (Invitrogen, 18064014) and random primers. The resulting cDNA was converted into dsDNA in the presence of dUTP to prevent subsequent amplification of the second strand and thus maintaining the ‘strandedness’ of the library. Following 3’ adenylation and adaptor ligation, libraries were subjected to 15 cycles of PCR to produce libraries ready for sequencing. The libraries were quantified on the Perkin Elmer LabChip GX with the DNA High Sensitivity Reagent kit (Perkin Elmer, CLS760672). Libraries were pooled in equimolar ratios, and the pool was quantified by qPCR using the KAPA Library Quantification Kit—Illumina/Universal (KAPA Biosystems, KK4824) in combination with the Life Technologies Viia 7 real time PCR instrument.

Bulk RNA-sequencing was performed using the Illumina NextSeq500 (NextSeq control software v2.1.0/Real Time Analysis v2.4.11). The library pool was diluted and denatured according to the standard NextSeq protocol (Document # 15048776 v02) and sequenced to generate paired-end 76 base pair reads using a 150 cycle NextSeq500/550 High Output reagent Kit v2 (Illumina, FC-404-2002). After sequencing, fastq files were generated using the bcl2fastq2 (v2.18.0, demultiplexed option used, available from Illumina) and received from the Institute for Molecular Bioscience Sequencing Facility (University of Queensland). Salmon (v1.2.0; validate mappings and gcBias options used) [8] was used for quantifying transcript abundance. The count data was loaded into R (v4.0.3) using tximeta (v1.8.3; default import option used) [9]. Differential gene expression analysis between *Slc13a4*^+/−^ and wild-type samples was carried out in R using the DeSeq2 pipeline (v1.30.0; estimate size factors and walt test options used) [10]. Gene expression levels between *Slc13a4*^+/−^ and wild-type samples were compared using Wald test as implemented in the DESeq2 pipeline. *p*-values were corrected using Benjamini–Hochberg adjustment. A statistically significant difference in gene expression between cohorts was represented by an adjusted *p*-value < 0.1. Furthermore, to stratify differentially expressed transcripts, a fold change cut-off of >  ± 1.2 was employed.

The data repositories where the work presented in this manuscript can be found are listed in Table 1. The raw sequencing files have also been lodged, and are available at GEO (Data set 1) [11]. Differential gene analysis revealed 90 differentially regulated genes in the forebrain of *Slc13a4*^+/−^ mice (Data file 2) [12]. Of these, 55 were upregulated, and 35 were down downregulated in comparison to controls (Data file 3) [13].

**Table 1:** Overview of data files

Label	Name of data file	File types (file extension)	Data repository and identifier (DOI or accession number)
Data file 1	Sequencing data from P20 wild-type and *Slc13a4* heterozygous mouse forebrains	CEL file	https://identifiers.org/geo:GSE171765 [11]
Data file 2	Differentially expressed genes P20 forebrain *Slc13a4*^+/−^ vs *Slc13a4*^+/+^	Excel spreadsheet (.xlsx)	https://doi.org/10.6084/m9.figshare.13088978 [12]
Data file 3	Volcano plot of differentially regulated genes in *Slc13a4* heterozygous mice	png	https://doi.org/10.6084/m9.figshare.14099321 [13]

## Limitations

This work complements our previously published work [5, 6] adding to our understanding of the critical pathways required during the postnatal period for normal brain development. Moreover we have attempted to identify those pathways functioning in the presence of elevated sulfate levels during this critical window demonstrating an important role for this sulfate transporter SLC13A4 in regulating brain development. There are a number of limitations to this work, however. Firstly our analysis of the transcriptomic changes in the forebrain of *Slc13a4*^+/−^ was only conducted at one postnatal age P20. Investigating consecutive ages during the early developmental window would provide further context around the genetic landscape that requires elevated sulfate levels for brain genesis. In turn, this could highlight common genes/pathways that may be essential drivers of normal brain development throughout this period. Secondly, although the expression of SLC13A4 is predominantly within the choroid plexus and pia mater of the forebrain at this age [5], our design was not based on a cell specific approach. Performing single cell RNA-sequencing in future could circumvent this limitation to pinpoint at a cell specific level the gene requirements for elevated sulfate levels to maintain normal brain development over the postnatal period.

## Data Availability

The raw sequencing data described in this Data note can be freely and openly accessed through GEO (Data file 1) [11]. Data file 2 and Data file 3 are available on Figshare (https://figshare.com) [12, 13]. Please see Table 1 for links to the data.

## Abbreviations

SLC13A4: Solute Carrier Family 13 (Sodium/Sulphate Symporters) Member 4
RNA-Seq: RNA-Sequencing
P: Postnatal day
PCR: Polymerase chain reaction
